# The drivers of month-of-birth differences in children's cognitive and non-cognitive skills

**DOI:** 10.1111/rssa.12071

**Published:** 2014-07-15

**Authors:** Claire Crawford, Lorraine Dearden, Ellen Greaves

**Affiliations:** Institute for Fiscal Studies London and University of WarwickCoventry, UK; Institute for Fiscal Studies London and Institute of EducationLondon, UK; Institute for Fiscal Studies London and University of WarwickCoventry, UK

**Keywords:** Age of starting school, Age at test, Month of birth, Non-cognitive skills

## Abstract

Previous research has found that children who are born later in the academic year have lower educational attainment, on average, than children who are born earlier in the year, especially at younger ages; much less is known about the mechanisms that drive this inequality. The paper uses two complementary identification strategies to estimate an upper bound of the effect of age at test by using rich data from two UK birth cohorts. We find that differences in the age at which cognitive skills are tested accounts for the vast majority of the difference in these outcomes between children who are born at different times of the year, whereas the combined effect of the other factors (age of starting school, length of schooling and relative age) is close to zero. This suggests that applying an age adjustment to national achievement test scores may be an appropriate policy response to overcome the penalty that is associated with being born later in the academic year. Age at test does not, however, explain all of the difference in children's view of their own scholastic competence. Age adjusting national achievement test scores may help to overcome differences in ability beliefs between children who are born at different times of the year, but our results suggest that additional policy responses may be required.

## 1. Introduction

Previous research has found that children who are born at the start of the academic year achieve better examination results, on average, than children who are born at the end of the academic year (e.g. Fredriksson and Öckert (2013), Bedard and Dhuey (2006), Datar (2006), Puhani and Weber (2007), Black *et al*. (2011), McEwan and Shapiro (2008) and Smith (2009)). This pattern is consistent across countries for children of younger ages, with the differences generally diminishing as children grow older (Crawford *et al*.,2007,^2013^; Robertson, 2011). In England, where the academic year runs from September 1st to August 31st, this means that children who are born in the autumn tend to outperform those who are born in the summer (e.g. Russell and Startup (1986), Sharp *et al*. (1994), Thomas (1995) and Alton and Massey (1998)).

Our own previous research (Crawford *et al.* 2007), based on administrative data held by the UK Department for Education, showed that August-born children in England score, on average, over half a standard deviation lower than their September-born counterparts in national achievement tests at age 7 years. This difference decreases over time but is still significant at age 16 years, when young people are making decisions about whether to stay on for post-compulsory education. At just over 10% of a standard deviation, this gap translates into a 5.8-percentage-point reduction in the likelihood that August-borns will reach the government's expected level of five General Certificates of Secondary Education at grades A^*^–C, which is usually regarded as the standard required to continue in post-compulsory education. By contrast, Robertson (2011) found that differences between those who are born earlier and later in the academic year are eliminated by the eighth grade (around ages 12–14 years) in a US sample, where ‘redshirting’ (or delaying a child's entry to school) is more common.

Crawford *et al*. (2010) also found evidence that August-borns are 1.5 percentage points less likely to continue into higher education at age 18 or 19 years than those who are born in September (see also Higher Education Funding Council for England (2005)). However, both Crawford *et al*. (2013) and Pellizzari and Billari (2012) found that performance at university is highest for those who are born *later* in the academic year, which is consistent with selection of the most able younger students into higher education, among other theories. For example, Crawford *et al*. (2013) found that those who are born at the end of the academic year are 2.5 percentage points more likely to complete their degree and 1 percentage point more likely to achieve a first- or upper second-class degree than those who are born at the start of the academic year, although this latter estimate is not significantly different from 0.

Given the importance of educational attainment in determining a range of later life outcomes, these differences mean that the month in which individuals are born (relative to the academic year cut-off in their jurisdiction) has the potential to affect them throughout their lives, with some researchers finding significant effects on wages (e.g. Bedard and Dhuey (2012), Fredriksson and Öckert (2013), Kawaguchi (2011) and Solli (2011)) and other adult outcomes (e.g. Du *et al*. (2012) and Muller and Page (2013)).

Recent evidence (e.g. Goodman *et al*. (2003), Crawford *et al*. (2007), Dhuey and Lipscomb (2008, 2010), Elder and Lubotsky (2009), Mühlenweg (2010) and Mühlenweg *et al*. (2012)) has also started to show that other outcomes that are observed during childhood might also differ by month of birth. This is important for at least two reasons: first, because they may affect children's wellbeing in the short term; for example, being among the youngest (and perhaps also the smallest) in your class may increase your chances of being bullied or lower your self-esteem; second, because they may have potentially serious long-term consequences for children's lives. For example, if continually being among the lowest academic performers in your class or school affects your motivation and determination to do well, or your belief in your ability to control your own destiny (locus of control), then the month in which you were born may have short- and longer-term consequences far beyond those captured by educational attainment alone.

Appropriate policy responses to the differences in attainment and wider outcomes for children who are born towards the start and end of the academic year depend on the mechanism through which these differences arise. This area of research has received less attention in the literature to date, in part because of the empirical challenges that identification presents, and it is to this literature that our paper contributes. We use two complementary identification strategies, in two sources of UK data, to make two contributions: first, we identify an upper bound on the contribution of a child's age when tested to the differences in attainment that we observe among children who are born at different times of the year in England. Second, we contribute the first exploration of the drivers of the differences in a range of non-cognitive outcomes, such as self-esteem and locus of control. These two contributions provide new insight into the types of policy responses that might be appropriate in helping to overcome the disadvantages that are faced by those who are born later in the academic year.

We hypothesize that there are four main potential drivers of the differences in outcomes between children who are born in different months of the year: first, in a system in which examinations are taken on a fixed date, as is the case in England, some children will be up to a year younger than others when they sit the tests (referred to as the ‘absolute age’ or ‘age-at-test’ effect). Second, those born just before the discontinuity may be disadvantaged by the fact that they started school considerably younger than their peers (the ‘age of starting school’ effect). Third, age relative to classroom or year group peers may adversely affect some children, e.g. if explicit comparison between children in the same class or year group negatively affects the appropriateness of the curriculum or self-belief of younger children (the ‘rank’ or ‘relative age’ effect). Finally, depending on the admissions system, some children who are born towards the end of the academic year may have attended school for fewer terms before the examination than those who are born towards the start of the academic year (the ‘length-of-schooling’ effect). We rule out season of birth as a likely explanation, as differences between children who are born at the start and end of the academic year are observed across a variety of jurisdictions in both the northern and the southern hemispheres which adopt a variety of academic year cut-offs (e.g. Australia, Chile, Japan, New Zealand and the USA).

Identifying which of these factors can explain the differences that we observe is challenging because, in jurisdictions where all children sit tests at the same time of year, there is an exact linear relationship between three of the four factors: 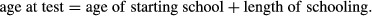
This means that it is not possible to identify separately the effect of each of these factors by using standard regression techniques, unless functional form restrictions are imposed (e.g. assuming that one effect is linear). Relative age is also highly correlated with absolute age: children who are the oldest when they sit the test also tend to be the oldest in their class or year group.

Some studies have overcome this difficulty by focusing on outcomes that are measured at around the same age for individuals beyond the end of compulsory schooling, thus breaking the perfect correlation between age at test and age at school entry. For example, Black *et al*. (2011) identified the effect of school starting age on intelligence quotient (IQ) scores taken as part of men's enrolment for military service at around age 18 years (as well as the likelihood of teenage pregnancy and earnings) by using Norwegian administrative data. They found that starting school younger has a small positive effect on IQ scores, as well as on the probability of teenage pregnancy. By contrast, they found a large and significant positive effect on IQ scores arising from sitting the test at an older age, suggesting that age at test is the main driver of differences between those who are born in different months of the year.

Other studies have attempted to separate these effects during compulsory education. For example, Datar (2006) relied on a functional form assumption to separate the age of starting school and the age-at-test (absolute age) effect, by assuming that the age-at-test effect is linear, i.e. that the difference in test scores between children who are 6 months apart in age is the same regardless of how old those children are. Under this (strong) assumption, and using the difference in pupils' test scores over time as the dependent variable, the effect of absolute age on test scores is differenced out, leaving only the age of starting school effect. Using data from the Early Childhood Longitudinal Study in the USA, Datar (2006) found that the test scores of older entrants increase by 0.12 standard deviations more than those of the youngest entrants over a 2-year period, implying that it is better for children to start kindergarten when they are older. There is no length-of-schooling effect (as all children enter kindergarten at the same time of year), but it is unclear whether or how the relative age effect features in her analysis.

Crawford *et al*. (2007) took advantage of the fact that school admissions policies in England are set by local, rather than central, authorities, meaning that there is considerable regional and temporal variation in the age at which children who are born on a particular day start school (and hence the amount of schooling that they receive before the tests). This identification strategy relies on making comparisons across areas and over time, which requires large sample sizes and means that it is very important to account for any differences across areas or cohorts that might affect test scores. Because the date on which children start school also dictates the number of terms of schooling that they receive before the test, it is not possible to separate the effect of starting school younger from the effect of receiving an additional term of schooling, although it is possible to separate the combination of these two effects from the age-at-test (absolute age) effect, which they did by imposing parametric assumptions.

Crawford *et al*. (2007) found that it is the age-at-test effect that matters most: at age 7 years, they found a small negative effect of starting school slightly older (and receiving one fewer term of schooling before the tests) of around 5% of a standard deviation; however, this effect is dwarfed by the age-at-test effect (which can be calculated—assuming linearity—by subtracting the combined age of starting school and length of schooling effects from the total effect) of around 60% of a standard deviation. Moreover, the age of starting school or length-of-schooling effect has disappeared completely by age 14 years.

Smith (2010) used an identification strategy that was very similar to that of Crawford *et al*. (2007), taking advantage of a temporary change in the school admissions policy in place in British Columbia to estimate an upper bound on the age-at-test effect and a lower bound on the age of starting school effect (neither of which can be separated from the length-of-schooling effect). Using administrative data on grade repetition at grade 3 (age 8–9 years) and literacy and numeracy scores at grade 10 (age 15–16 years), he found results very similar to those of Crawford *et al*. (2007): relatively large age-at-test effects and relatively small age of starting school effects.

This paper will add to the existing literature on the drivers of month-of-birth differences in educational attainment by using two complementary identification strategies which do not rely on temporal or regional variation in school admissions policies to identify an upper bound on the age-at-test effect. It will also provide new evidence on the extent to which a combination of the age of starting school, length-of-schooling and relative age effects can help to explain the differences in a range of non-cognitive skills between children who are born at the start and end of the academic year. These two complementary pieces of evidence are vital in providing a more complete picture of how best to respond to the differences in outcomes between children who are born at the start and end of the academic year.

This paper now proceeds as follows: Section Methodology and identifying assumptions discusses our identification strategies and the methods that were used to implement them. Section Data describes the data that we use. Section Results presents our results. Section Conclusions concludes.

## 2. Methodology and identifying assumptions

The academic year in England runs from September 1st to August 31st and is split into three terms. It is a statutory requirement for children to start school by the beginning of the term after they turn 5 years of age, but within these confines school admissions policies are set by local (rather than central) authorities, and in most cases children start school considerably earlier than this, often in the September after they turn 4 years of age.

Our aim is to identify the effect on a range of cognitive and non-cognitive skills of being born just after rather than just before the academic year cut-off of September 1st, i.e. at the start rather than the end of the academic year in England. This problem can be thought of as an experiment, where the ‘treatment’ is being the oldest in the academic year. Following standard notation we denote potential outcome variables under treatment and no treatment as *Y*_1_ and *Y*_0_ respectively. The evaluation problem arises because pupils are born at either the start or end of the academic year—they either do or do not receive the treatment—and hence it is impossible to observe both *Y*_1_ and *Y*_0_ for any given individual.

Many evaluation techniques have been developed to address this problem, which usually involve the construction of an appropriate control group whose outcomes represent the counterfactual outcomes for those in the treatment group (see Blundell and Costa Dias (2009) for a recent review). Regression discontinuity design (RDD) is often regarded as the quasi-experimental technique that comes closest to the experimental ‘gold standard’ (the randomized experiment) in appropriate applications (Lee and Lemieux, 2010). RDD provides a way of identifying mean treatment effects for a subgroup of the population (close to the discontinuity) under minimal assumptions (Hahn *et al.*, 2001). For example, parametric assumptions are not necessary, and the requirement to choose appropriate control variables (and their functional form) is removed. The limitation of RDD in some circumstances is that identification is relevant only for a subsection of the population (close to the discontinuity), but in many cases, including this one, this identifies a policy relevant parameter.

RDD has the defining characteristic that the probability of receiving the treatment changes discontinuously as a function of one or more underlying variables. Under certain conditions, which are detailed below, the allocation of treatment on the basis of this underlying variable is analogous to assignment in a randomized experiment, and the causal effect of the treatment at the point of discontinuity is recovered. In our application, the probability of receiving treatment (being the oldest in the academic year) is determined by date of birth (*Z*) and varies discontinuously at August 31st–September 1st. If the class application rule is strictly applied, those who are born on August 31st are the youngest in the academic year, whereas those who are born on September 1st are the oldest and hence receive the treatment. We denote treatment status by the binary variable *T*, where *T*=1 denotes treatment and *T*=0 denotes no treatment.

Following Hahn *et al*. (2001), to identify the causal effect of the treatment by using RDD we require the following conditions to be met.

(a) *The probability of treatment must vary discontinuously at some point with respect to Z*. Formally: 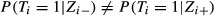, where *Z*_*i*−_ refers to the region that is immediately below the discontinuity and *Z*_*i*+_ immediately above. We argue that this condition holds in our application: although it is possible, in principle, for parents to request that their child starts school later than the local authority admissions policy would suggest, recent guidance issued by the UK Department for Education suggests that this is not happening in practice. Moreover, in contrast with many other countries, it is very rare for children in England to be held back a grade once they have started school. For example, in a census of state school pupils in England in 2008, over 99% of pupils were in the ‘correct’ academic year based on their age; 99.27% of those in the first year of primary school, falling only slightly to 99.12% for pupils at the end of compulsory schooling. In the first year of primary school the proportion in the correct year group is similarly high for those to the left and right of the discontinuity: 98.13% and 99.84% respectively. This means, for example, that there is almost a year's difference in the average age at which children who were born just before and just after the academic year cut-off start school or sit national achievement tests (Table 1).
(b) *Pupils' characteristics (aside from date of birth) must be continuous at the point of discontinuity*. Formally: 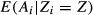 is continuous in *Z* at the discontinuity, where *A*_*i*_ represents all characteristics of pupils or their families that affect the outcome of interest. This assumption ensures that other factors are not responsible for any differences in outcomes observed between the treatment and non-treatment groups. We present a selection of evidence illustrating that there are no other obvious or significant discontinuities in parent or child characteristics at the point of discontinuity in 1Tables 2–4 (and discussed in more detail below).
(c) *Individuals do not select into the treatment on the basis of anticipated gains from treatment*. The fact that over 99% of children in England are in the correct academic year given their age—and that this proportion is only marginally lower among those who were born just before (99.0%) rather than just after (99.7%) the academic year cut-off—suggests that there is no strong evidence that those who are born towards the end of the academic year are being held back to take advantage of starting school (and sitting the tests) up to a year older.

**Table 1:** RDD estimates: age at test†

	*ALSPAC ‘Focus at*	*National assessment*
	*8 clinic' (days)*	*at age 6 or 7 years*
		*(key stage 1) (days)*
Treatment effect	12.526	346.661‡
	(22.969)	(13.510)
Distance	Yes	Yes
Distance × treatment	Yes	Yes
Distance^2^	Yes	Yes
Distance^2^ × treatment	Yes	Yes
Background characteristics	No	No
*N*	982	912
*R*^2^	0.004	0.814

† Standard errors (in parentheses) are robust and clustered by distance to the discontinuity. ‘Distance’ refers to the assignment variable: the distance from the discontinuity. The ALSPAC ‘Focus at 8 clinic’ refers to the date that the child attended the Avon Longitudinal Study of Parents and Children session around the child's eighth birthday.

‡ *p* < 0.001.

**Table 2:** Background characteristics of those born in August and September in the Avon Longitudinal Study of Parents and Children: RDD estimates†

*Characteristic*	*Treatment*	*Standard*	*N*	*R*^2^
	*effect*	*error*		
Male: no	0.065	0.078	982	0.011
Male: yes	−0.065	0.078	982	0.011
Household income quintile: lowest	0.013	0.064	982	0.004
Household income quintile: 2nd lowest	0.017	0.062	982	0.003
Household income quintile: 3rd lowest	−0.181‡	0.080	982	0.008
Household income quintile: 2nd highest	0.164‡	0.067	982	0.008
Household income quintile: highest	−0.046	0.073	982	0.002
Household income quintile: missing	0.033	0.095	982	0.003
Mother's age at birth: <20 years	−0.021	0.026	982	0.009
Mother's age at birth: 20≤*X*<25 years	−0.014	0.056	982	0.008
Mother's age at birth: 25≤*X*<30 years	−0.011	0.076	982	0.023
Mother's age at birth: 30≤*X*<35 years	0.073	0.083	982	0.004
Mother's age at birth: *X*⩾35 years	−0.044	0.070	982	0.005
Mother's age at birth: missing	0.016	0.027	982	0.001
Non-white: no	−0.023	0.045	982	0.001
Non-white: yes	0.013	0.021	982	0.009
Non-white: missing	0.010	0.039	982	0.013
English as additional language: no	−0.034	0.039	982	0.010
English as additional language: yes	0.024	0.017	982	0.002
English as additional language: missing	0.010	0.039	982	0.013
Household status: married	−0.080	0.078	982	0.008
Household status: cohabiting	0.042	0.052	982	0.008
Household status: lone parent	−0.014	0.023	982	0.004
Household status: missing	0.051	0.050	982	0.004
Father in work: no	0.034	0.033	982	0.005
Father in work: yes	−0.009	0.078	982	0.005
Father in work: missing	−0.025	0.076	982	0.005
Mother in work: no	−0.038	0.083	982	0.005
Mother in work: yes	0.024	0.088	982	0.001
Mother in work: missing	0.014	0.080	982	0.007
Mother's education: Certificate of Secondary Education	−0.010	0.073	982	0.004
Mother's education: vocational	−0.089	0.050	982	0.006
Mother's education: O level	0.038	0.086	982	0.002
Mother's education: A level	0.099	0.086	982	0.005
Mother's education: degree	−0.048	0.053	982	0.003
Mother's education: missing	0.011	0.037	982	0.001
Father's education: Certificate of Secondary Education	−0.075	0.098	982	0.003
Father's education: vocational	0.000	0.081	982	0.004
Father's education: O level	0.041	0.085	982	0.003
Father's education: A level	−0.078	0.073	982	0.003
Father's education: degree	0.074	0.077	982	0.003
Father's education: missing	0.038	0.036	982	0.002
Mother's social class: i	0.015	0.035	982	0.001
Mother's social class: ii	−0.066	0.102	982	0.004
Mother's social class: iii (manual)	0.027	0.113	982	0.001
Mother's social class: iii (non-manual)	0.025	0.048	982	0.004
Mother's social class: iv	0.017	0.047	982	0.006
Mother's social class: v	−0.013	0.009	982	0.004
Mother's social class: missing	−0.004	0.073	982	0.003
Father's social class: i	0.041	0.059	982	0.009
Father's social class: ii	0.062	0.091	982	0.006
Father's social class: iii (manual)	0.006	0.065	982	0.003
Father's social class: iii (non-manual)	0.020	0.084	982	0.003
Father's social class: iv	−0.065	0.057	982	0.005
Father's social class: v	−0.027	0.046	982	0.004
Father's social class: missing	−0.036	0.067	982	0.007
Ever lived in social housing: no	−0.021	0.067	982	0.004
Ever lived in social housing: yes	0.012	0.066	982	0.003
Ever lived in social housing: missing	0.009	0.013	982	0.003
Own or mortgage home: no	0.138	0.079	982	0.003
Own or mortgage home: yes	−0.147	0.077	982	0.003
Own or mortgage home: missing	0.009	0.013	982	0.003
Financial difficulties: no	−0.008	0.110	982	0.001
Financial difficulties: yes	−0.005	0.093	982	0.001
Financial difficulties: missing	0.013	0.029	982	0.001
Breastfed: no	0.004	0.068	982	0.009
Breastfed: yes	−0.018	0.068	982	0.009
Breastfed: missing	0.014	0.028	982	0.002
Smoke around child: no	−0.109	0.097	982	0.004
Smoke around child: yes	0.117	0.083	982	0.004
Smoke around child: missing	−0.008	0.058	982	0.003
Multiple birth: no	0.008	0.040	982	0.006
Multiple birth: yes	−0.008	0.040	982	0.006
Number older siblings: 0	−0.066	0.111	982	0.011
Number older siblings: 1	0.056	0.085	982	0.009
Number older siblings: 2	−0.075	0.059	982	0.005
Number older siblings: ⩾3	0.065	0.055	982	0.009
Number older siblings: missing	0.020	0.055	982	0.005

† Standard errors are robust and clustered by distance to the discontinuity. ‘Distance’ refers to the assignment variable: the distance from the discontinuity. All specifications include an interaction between the distance to the discontinuity and treatment indicator, and interaction between distance squared and the treatment indicator. No additional background characteristics are included in the model.

‡ *p*< 0.05.

**Table 3:** Background characteristics of those born in August and September in the Avon Longitudinal Study of Parents and Children: seemingly unrelated regression joint significance tests—*p*-values†

*Characteristic included*	*p-values for the following windows:*			
	*Window of*	*Window of*	*Window of*	*Window of*
	*10 days*	*20 days*	*30 days*	*40 days*
Household income quintile	0.97	0.71	0.56	0.54
*Plus* mother's age at birth	0.45	0.32	0.7	0.28
*Plus* whether non-white	0.62	0.48	0.82	0.29
*Plus* whether English as additional language	0.65	0.42	0.74	0.3
*Plus* household's marital status	0.63	0.29	0.8	0.41
*Plus* whether mother in work	0.73	0.26	0.87	0.45
*Plus* whether father in work	0.77	0.35	0.92	0.5
*Plus* mother's education	0.62	0.47	0.97	0.67
*Plus* father's education	0.6	0.46	0.84	0.63
*Plus* mother's social class	0.65	0.56	0.88	0.72
*Plus* father's social class	0.51	0.5	0.92	0.81
*Plus* whether ever lived in social housing	0.27	0.55	0.93	0.74
*Plus* whether always owned or mortgage home	0.26	0.26	0.84	0.74
*Plus* financial difficulties reported	0.29	0.31	0.88	0.79
*Plus* whether child was breastfed	0.32	0.36	0.91	0.84
*Plus* whether anyone smokes around child	0.31	0.31	0.87	0.78
*Plus* child's birth weight	0.19	0.33	0.9	0.82
*Plus* whether multiple birth	0.17	0.37	0.93	0.86
*Plus* number of older siblings	0.15	0.24	0.85	0.67

† *p*-values from joint significance tests from seemingly unrelated regressions are reported. The first row presents the significance test when only household income quintile is included. Subsequent rows add further characteristics of the household and child.

**Table 4:** McCrary density test: log-difference in frequency bins†

*Window size*	*Results for the following bin sizes:*			
	*1*	*2*	*3*	*4*
20	−0.22	−0.42	−0.29	−0.27
	(0.28)	(0.23)	(0.48)	(0.49)
30	−0.34	−0.42	−0.31	−0.34
	(0.20)	(0.21)	(0.23)	(0.27)
40	−0.33	0.43‡	−0.34	−0.36
	(0.19)	(0.19)	(0.22)	(0.21)

† Coefficients and standard errors (in parentheses) are presented for alternative window and bin sizes. A common sample is imposed. The McCrary density test was implemented in Stata by using the user-written command available here: http://emlab.berkeley.edu/∼jmccrary/DCdensity/.

‡ *p* < 0.05.

The other dimension on which individuals could potentially select into treatment is on the basis of their date of birth. Although a pupil has no power to manipulate their date of birth, parents have some means to manipulate the month in which their child is born (either through conception or birth decisions). Some studies have found systematic differences in the number (e.g. Gans and Leigh (2009)) or family background characteristics (e.g. Buckles and Hungerman (2013)) of children who are born on either side of the discontinuity, which might result from sorting of this kind. However, we find no significant evidence of this in our sample: Table 2 presents RDD estimates where the outcome variable is a household or child attribute. Although a small minority of these differences are large, there are significant differences in fewer than 5% of cases. Moreover, the differences do not all point in the same direction (for example they do not suggest that children who are born just after the discontinuity are systematically more likely to come from more educated or affluent families than those who are born just before the discontinuity). Results from a seemingly unrelated regression of all parent and child attributes, which are presented in Table 3, also suggest that there are no systematic differences in the immediate vicinity of the discontinuity.

**Fig 1:**
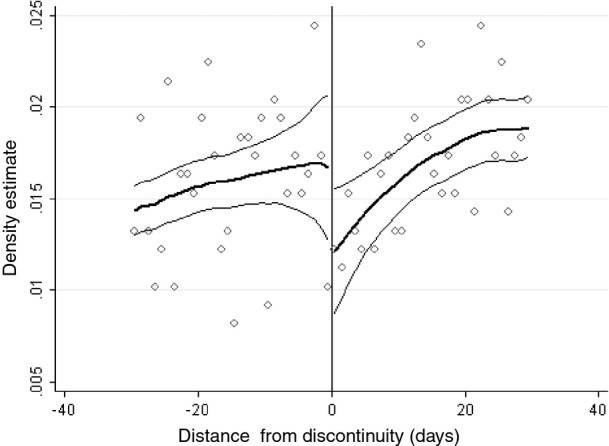
McCrary density test: a window of 30 days on either side of the discontinuity is applied, with a bandwidth of 1; both discontinuities (across cohorts) have been pooled to increase sample sizes; a common sample is imposed; the McCrary density test was implemented in Stata by using the user-written command that is available here: http://emlab.berkeley.edu/∼jmccrary/DCdensity/

McCrary density tests (McCrary, 2008), which are presented in Table4, also suggest that there is no systematic change in the density of births on either side of the discontinuity, for a range of specified bin and window sizes. Fig. 1 presents a graphical representation of the results from the McCrary density test when a bin size of 1 and a window size of 30 days is imposed. There appears to be a slightly lower density of births to the right of the discontinuity, although this difference is not significant, and goes in the opposite direction to that which would be suggested by the sorting hypothesis that was outlined above.

Taken together, these results provide no strong evidence that parents or pupils are selecting into the treatment on the basis of anticipated gains from doing so.

Given the very small numbers of pupils who do not start school or sit the tests at the expected time, we argue that our application is a ‘sharp’ discontinuity. When the discontinuity is sharp, the average causal effect of being the oldest in the academic year for those around the discontinuity at 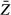 (under the assumptions above) is given by 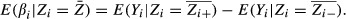
1

The necessary assumptions are more likely to hold in a small region around the discontinuity, 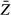, suggesting that a small window should be used. Including observations from a larger region increases the sample size, however, which implies a trade-off between statistical power and the calculation of unbiased estimates.

The average causal effect can be estimated non-parametrically (e.g. by using local linear non-parametric or kernel regression), or parametrically, by approximating the continuous underlying function of *Z* by using a polynomial of varying degrees. Our main specification controls parametrically for distance to the discontinuity by using a quadratic polynomial which we allow to vary on either side of the discontinuity.

Although Tables 4 provide little evidence of any systematic differences in child or household attributes on either side of the discontinuity, to improve the precision of our estimates, we include a range of individual and family background characteristics in our models. Froelich (2007) and others have shown that the identifying assumptions underlying regression discontinuity analysis hold in the presence of additional covariates.

Our final RDD specification thus has the form 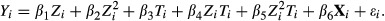
2

For each individual *i*, *Y*_*i*_ represents their outcome of interest. *T*_*i*_ is a binary variable that represents the treatment of being born after the discontinuity; it is equal to 1 if a child is among the oldest in the academic year and 0 if they are among the youngest. *Z*_*i*_ represents the distance from the discontinuity: the ‘assignment variable’ that was referred to above. For example, *Z*_*i*_ is equal to 0 if cohort member *i* was born on September 1st, 1 if the cohort member was born on September 2nd and −1 if the cohort member was born on August 31st. The addition of the squared term 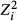 allows the effect of a child's date of birth to affect their outcome non-linearly. As stated above, we assume that a child's date of birth has a smooth effect on their outcomes, and that in the absence of a discontinuity on September 1st there would be no ‘jump’ in outcomes. The inclusion of interaction terms *Z*_*i*_*T*_*i*_ and 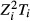 allows the effect of the assignment variable to vary on either side of the discontinuity to increase the flexibility of the specification. **X**_*i*_ represents a vector of observable pupil and parent characteristics that may affect the outcomes of interest (which are described in more detail below), and *ɛ*_*i*_ represents unobservable and random factors that may also affect these outcomes.

For comparability with previous work which focuses on children's month of birth, our preferred window is 30 days either side of the discontinuity. Cross-validation to determine the optimal bandwidth suggests that no window around the discontinuity should be imposed, but our results are robust to this alternative specification. As described above, we choose to use a quadratic specification for the assignment variable—but our results are robust to alternative choices of window size and higher or lower order polynomials (Tables5 and 6). We also investigate the assumption that the assignment variable is smooth in the absence of a discontinuity by running a series of placebo tests (Tables9). Following Card and Lee (2008), we cluster standard errors by day of birth (as was also done by Berlinski *et al*. (2009), Brewer and Crawford (2010) and Fitzpatrick (2012)), but the overall significance of our estimates does not change if we instead cluster standard errors on the basis of school attended.

**Table 5:** Robustness checks for cognitive outcomes†

*Specification*	*Results for KS 1*			*Results for WISC*			*Results for WISC, correlation above 0.3*			*Results for WISC, correlation above 0.4*			*Results for WOLD, comprehension*			*Results for WOLD, expression*		
	*Treatment effect*	*Standard error*	*N*	*Treatment effect*	*Standard error*	*N*	*Treatment effect*	*Standard error*	*N*	*Treatment effect*	*Standard error*	*N*	*Treatment effect*	*Standard error*	*N*	*Treatment effect*	*Standard error*	*N*
(1) Baseline	0.835‡	0.219	982	0.065	0.191	982	0.046	0.179	982	−0.029	0.181	982	−0.127	0.254	982	0.050	0.162	982
(2) Exclude covariates	0.876‡	0.188	982	0.101	0.205	982	0.089	0.188	982	−0.011	0.191	982	−0.170	0.217	982	0.075	0.156	982
(3) No common sample	0.659‡	0.139	3026	0.109	0.162	1359	0.026	0.154	1404	−0.028	0.163	1403	−0.105	0.203	1385	0.081	0.124	1384
(4) At least one ALSPAC outcome	0.631§	0.227	1202	—			—			—			—			—		
(5) Window size: 20	0.697§	0.273	0	0.124	0.250	657	0.145	0.237	657	0.011	0.244	657	0.016	0.353	657	0.112	0.208	657
(6) Window size: 40	0.767‡	0.188	1293	0.042	0.159	1293	0.007	0.146	1293	−0.019	0.150	1293	−0.010	0.211	1293	0.104	0.146	1293
(7) Window size: 180	0.722‡	0.077	5081	−0.33	0.079	5081	−0.087	0.077	5081	−0.036	0.086	5081	0.018	0.088	5081	0.014	0.078	5081
(8) Polynomial: degree 1	0.772‡	0.135	982	0.041	0.124	982	0.001	0.122	982	0.006	0.131	982	0.116	0.162	982	0.059	0.133	982
(9) Polynomial: degree 3	0.587‡	0.315	982	0.126	0.250	982	0.142	0.241	982	0.025	0.259	982	−0.078	0.372	982	0.040	0.228	982
(10) Cluster standard errors at the school level	0.835‡	0.196	982	0.065	0.199	982	0.046	0.182	982	−0.029	0.160	982	−0.127	0.218	982	0.050	0.178	982
(11) Common trend across discontinuity	0.773‡	0.134	982	0.042	0.124	982	0.002	0.122	982	0.008	0.131	982	0.115	0.164	982	0.059	0.133	982
(12) 2-day bins	0.833‡	0.222	982	0.055	0.202	982	0.043	0.188	982	−0.013	0.192	982	−0.121	0.255	982	0.055	0.174	982
(13) Control for date of interview	—			0.058	0.184	982	0.037	0.171	982	−0.039	0.172	982	−0.138	0.241	982	0.039	0.163	982
(14) Exclude those closest to the discontinuity	1.444‡	0.294	820	−0.400	0.376	820	−0.355	0.346	820	−0.164	0.428	820	−0.230	0.386	820	0.114	0.402	820

† Standard errors are robust and clustered by distance to the discontinuity, except in specification (9) where standard errors are clustered at the school level. All specifications include background characteristics as additional covariates, except specification (2). All specifications include the distance from the discontinuity and distance squared, both interacted with the treatment indicator except specifications (8) and (9): specification (8) excludes the squared terms and specification (9) includes cubed terms. The window around the discontinuity is 30 days, unless otherwise specified (specifications (5), (6) and (7)). ‘Distance’ refers to the assignment variable: the distance from the discontinuity. ‘Wechsler intelligence scale for children’ (WISC) components included where the correlation in the whole sample is above 0.3 are information, similarities, arithmetic, vocabulary, digit span, backward digit span and coding. WISC components included where the correlation in the whole sample is above 0.4 are information, arithmetic and vocabulary. Row (4) is not applicable for outcomes that are observed in the Avon Longitudinal Study of Adults and Children (ALSPAC), as the sample is the same as that in row (3) for ALSPAC outcomes; if the ALSPAC outcome is observed (to appear when no sample restrictions are applied) by definition at least one ALSPAC outcome must be observed, so that the sample criterion is met. WOLD, ‘Wechsler objective language dimensions’.

‡ *p* < 0.001.

§ *p* < 0.01.

§§ *p* < 0.05.

**Table 6:** Robustness checks for non-cognitive outcomes†

*Specification*	*Results for scholastic competence*			*Results for likes school very much*			*Results for locus of control*			*Results for self-esteem*		
	*Treatment effect*	*Standard error*	*N*	*Treatment effect*	*Standard error*	*N*	*Treatment effect*	*Standard error*	*N*	*Treatment effect*	*Standard error*	*N*		
(1) Baseline	0.557‡	0.180	982	0.051	0.181	982	−0.028	0.192	982	0.042	0.222	982
(2) Exclude covariates	0.599§	0.155	982	0.068	0.153	982	−0.012	0.169	982	0.077	0.215	982
(3) No common sample	0.373§§	0.165	1308	−0.007	0.144	1353	0.101	0.164	1285	0.033	0.186	1308
(4) At least one ALSPAC outcome												
(5) Window size: 20	0.505§§	0.238	657	0.023	0.210	657	−0.170	0.264	657	0.109	0.271	657
(6) Window size: 40	0.411‡	0.153	1293	0.099	0.147	1293	0.097	0.152	1293	0.015	0.194	1293
(7) Window size: 180	0.181§§	0.083	5081	0.030	0.078	5081	−0.005	0.081	5081	0.014	0.078	5081
(8) Polynomial: degree 1	0.348‡	0.124	982	0.055	0.125	982	0.194	0.120	982	−0.036	0.146	982
(9) Polynomial: degree 3	0.744‡	0.242	982	−0.003	0.222	982	−0.104	0.251	982	0.249	0.280	982
(10) Cluster standard errors at the school level	0.557‡	0.193	982	0.051	0.217	982	−0.028	0.172	982	0.042	0.214	982
(11) Common trend across discontinuity	0.347‡	0.125	982	0.053	0.125	982	0.192	0.123	982	−0.037	0.147	982
(12) 2-day bins	0.565‡	0.196	982	0.024	0.191	982	−0.040	0.206	982	0.029	0.237	982
(13) Control for date of interview	0.562‡	0.180	982	0.053	0.181	982	−0.034	0.191	982	0.053	0.222	982
(14) Exclude those closest to the discontinuity	0.361	0.383	820	0.186	0.348	820	0.103	0.373	820	−0.272	0.372	820

† Standard errors are robust and clustered by distance to the discontinuity, except in specification (9) where standard errors are clustered at the school level. All specifications include background characteristics as additional covariates, except specification (2). All specifications include the distance from the discontinuity and distance squared, both interacted with the treatment indicator except specifications (8) and (9): specification (8) excludes the squared terms and specification (9) includes cubed terms. The window around the discontinuity is 30 days, unless otherwise specified (specifications (5), (6) and (7)). ‘Distance’ refers to the assignment variable: the distance from the discontinuity. Wechsler intelligence scale for children components included where the correlation in the whole sample is above 0.3 are information, similarities, arithmetic, vocabulary, digit span, backwards digit span and coding. Wechsler intelligence scale for children components included where the correlation in the whole sample is above 0.4 are information, arithmetic and vocabulary. Row (4) is not applicable for outcomes observed in the Avon Longitudinal Study of Parents and Children as the sample is the same as that in row (3) for ALSPAC outcomes; if the ALSPAC outcome is observed (to appear when no sample restrictions are applied) by definition at least one ALSPAC outcome must be observed, so that the sample criterion is met.

‡ *p* < 0.01.

§ *p* < 0.001.

§§ *p* < 0.05.

**Table 7:** RDD estimates: educational attainment at age 7 years; placebo tests with discontinuity at the first day of each month†

*Date*	*Estimates for window of 30 days*		
	*Treatment*	*Standard*	*N*
	*effect*	*error*	
September 1st	0.835‡	0.219	982
October 1st	0.112	0.135	998
November 1st	0.152	0.134	972
December 1st	0.117	0.142	922
January 1st	−0.102	0.235	734
February 1st	0.065	0.307	514
March 1st	−0.081	0.235	506
April 1st	0.011	0.186	737
May 1st	−0.060	0.161	938
June 1st	−0.184	0.204	960
July 1st	−0.068	0.171	988
August 1st	−0.162	0.157	985

† Standard errors are robust and clustered by distance to the discontinuity.

‡ *p* < 0.001.

**Table 8:** RDD estimates: Wechsler intelligence scale for children at age 8 years; placebo tests with discontinuity at the first day of each month†

	*Estimates for window of 30 days*		
	*Treatment effect*	*Standard error*	*N*
September 1st	0.065	0.191	982
October 1st	−0.094	0.120	998
November 1st	0.206	0.157	972
December 1st	0.221	0.171	922
January 1st	−0.227	0.192	734
February 1st	0.179	0.188	514
March 1st	−0.166	0.298	506
April 1st	−0.099	0.291	737
May 1st	−0.088	0.197	938
June 1st	−0.329‡	0.157	960
July 1st	−0.098	0.158	988
August 1st	−0.085	0.160	985

† Standard errors are robust and clustered by distance to the discontinuity.

‡ *p* < 0.05.

**Table 9:** RDD estimates: scholastic competence at age 8 years; placebo tests with discontinuity at the first day of each month†

*Date*	*Estimates for window of 30 days*		
	*Treatment*	*Standard*	*N*
	*effect*	*error*	
September 1st	0.557‡	0.180	982
October 1st	0.126	0.204	998
November 1st	−0.253	0.198	972
December 1st	−0.153	0.158	922
January 1st	−0.346	0.217	734
February 1st	0.846‡	0.259	514
March 1st	0.176	0.227	506
April 1st	−0.051	0.242	737
May 1st	−0.203	0.184	938
June 1st	0.156	0.219	960
July 1st	−0.004	0.160	988
August 1st	−0.172	0.271	985

† Standard errors are robust and clustered by distance to the discontinuity.

‡ *p* < 0.01.

The treatment effect of being the oldest in the academic year is represented by *β*_3_. From the discussion in Section Introduction, it is clear that *β*_3_ is a function of four potential mechanisms: age at test (age_*i*_), age of starting school (agess_*i*_), length of schooling (length_*i*_) and relative age (relage_*i*_). When tests are taken at the same point in time, it is possible that all four effects are contributing to the month-of-birth differences that we observe.

*Case 1*: 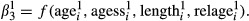
3In the case where individuals on either side of the discontinuity are tested at the same age 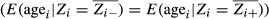, the treatment effect is a function of three potential mechanisms only, as age at test is eliminated.

*Case 2*: 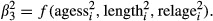
4

Case 2 therefore provides an assessment of the extent to which the combined age of starting school, length of schooling and relative age effects contribute to the differences in outcomes between children who are born just before and just after the academic year cut-off.

The comparison between 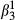 and 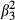
—i.e. the treatment effects when tests are taken at the same age (2) *versus*. the same point in time (1)—instead provides an indication of the extent to which age at test contributes to the treatment effect of being the oldest in the academic year. For this comparison to be informative about the magnitude of the age-at-test effect, the key requirement is for each of the four factors of interest—age at test, age of starting school, length of schooling and relative age—to have the same effects on the outcomes measured at the same age or at the same point in time. We use two complementary strategies (which are described in more detail below) to try to achieve this.

However, it must be noted that subtracting the difference in test scores when children are assessed at the same age (case 2) from the difference in test scores when children are assessed at the same time of year (case 1) does not isolate the age-at-test effect. Although the difference in relative age and age of starting school between those who are born just before and just after the discontinuity should be the same regardless of whether tests are taken at the same age or at the same point in time, the difference in length of schooling before the test will vary. When children start school at the same time of year (in the September after they turn 4 years of age in England) and are tested at the same age, those who were born at the start of the academic year will typically have had *less* schooling at the time of the test than those who were born at the end of the academic year (because they tend to start school older). Under the assumption that length of schooling has a positive effect on test scores (and that the effect of age at test is separable), the comparison between 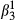 and 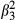 will therefore be an upper bound on the effect of age at test.

As described above, the comparison of the estimated treatment effect under case 1 and case 2 relies on the fundamental assumption that the tests taken in both cases are sufficiently comparable that the age of starting school and relative age effects cancel out. Ideally, the same test would be available in both case 1 and case 2, but we are aware of no source of UK data that contains test score information of this kind.

We address this problem in two ways: first we make careful comparisons across tests, ensuring that the components of the tests that we use in our analysis are as similar as possible; we also present estimates for alternative available tests.

Second, we adopt a complementary methodology as a robustness check on our main RDD results. Here, we make use of a different data set in which, under the imposition of certain parametric assumptions, we can use the same assessment to estimate the treatment effect in case 1 and case 2. (We cannot replicate this method of analysis in our main data set, as we do not observe sufficient overlap in age or month of interview among children who are born in different months of the year to identify the relevant effects, even relying heavily on linear extrapolation.) As this alternative data set focuses on a sample of children who were born in a single academic year—meaning that an RDD is not feasible—we instead use a linear regression model to estimate the change in the treatment (in this case being born in September relative to August, rather than being born marginally to the right rather than marginally to the left of the discontinuity) under case 1 and case 2.

In case 1, we estimate the equation below, where MOB_*im*_ represents child *i*'s month of birth, and MOI_*i*_ represents the month in which child *i* was tested. The inclusion of the linear variable MOI_*i*_ replicates the situation in which tests are taken in the same month, under the assumption that the month of interview is independent of a child's underlying cognitive ability, conditional on their month of birth and other observable characteristics included in the vector **X**_*i*_: 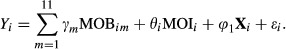
5

In case 2, we estimate the equation below, where the linear variable MOI_*i*_ has been omitted. Instead, a linear variable for the child's age at test (AGE_*i*_) is included to replicate the situation where tests are taken when children reach a particular age, under the assumption that age at test is independent of a child's underlying cognitive ability, conditional on their month of birth and other observable characteristics that are included in the vector **X**_*i*_: 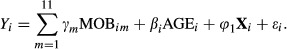
6

It is worth noting that the treatment effect that is identified by using our parametric regression approach is not the same as the treatment effect that is identified by using an RDD, as the RDD estimate pinpoints the effect of the discontinuity itself (i.e. of being born just after rather than just before the academic year cut-off), whereas here we are estimating the effect of being born in one month relative to another. It is also worth noting that our parametric regression approach is identified by using within- rather than between-cohort variation, although previous evidence on this topic (e.g. Crawford *et al*. (2007)) suggests that this does not matter markedly for our results.

The validity of these assumptions is discussed in Section Data.

## 3. Data

We use two of the UK's rich birth cohort studies to carry out our analysis: the Avon Longitudinal Study of Parents and Children (ALSPAC) and the Millennium Cohort Study (MCS).

### 3.1. Avon Longitudinal Study of Parents and Children

ALSPAC is a longitudinal study that has followed the children of around 14000 pregnant women whose expected date of delivery fell between April 1st, 1991, and December 31st, 1992, and who were resident in the Avon area of South-West England at that time. This means that ALSPAC cohort members are spread across three academic years, including children who were born on either side of two separate academic year discontinuities (around September 1st, 1991, and September 1st, 1992).

ALSPAC cohort members and their families have been surveyed via high frequency postal questionnaires from the time of pregnancy onwards, with information collected on a wide range of family background characteristics. The cohort members' cognitive and non-cognitive skills have also been assessed at various points throughout childhood via a series of clinic sessions. In addition, cohort members have been linked to their scores in national achievement tests at ages 7, 11, 14 and 16 years. The admissions policies that were in place in Avon during the period in which ALSPAC cohort members started school suggested that the vast majority of children in the survey would have been expected to start school in the September after they turned 4 years of age.

Our sample consists of the 4668 young people who were born in all months of the year for whom we observe all relevant outcomes (although the number who were born in various windows around each of our two discontinuities is obviously much smaller than this). Our final sample is smaller than the full ALSPAC sample for two main reasons: there was considerable attrition from the survey between birth and age 8 years (only 7488 young people were present at this latter age) and there is also some item non-response. This means that the number of observations for each survey outcome ranges between 6794 and 7439, with just 4668 young people having all survey outcomes present. The requirement to observe national assessment data does not further diminish our sample. It should be noted, however, that our results are robust to alternative sample definitions (presented in Tables5 and 6).

For our identification of an upper bound of the age-at-test effect on cohort members' cognitive skills, we compare scores from national achievement tests taken during the academic year in which a child turns 7 years of age (on the same date for children in the same academic cohort) with a measure of the cohort members' IQ taken during a clinic session around their eighth birthday. In contrast with tests taken in school, there is no significant difference in the age at which the IQ test was sat (see Table1).

As part of the national achievement tests, pupils were assessed on the basis of reading, writing and mathematics. We sum the scores that are available from all three subjects and use the total points score as our measure of cognitive development. We standardize each score according to the mean and standard deviation of that test in our sample, to have mean 0 and standard deviation 1.

The measure of cognitive development that is used in the survey is the third version of the ‘Wechsler’ intelligence scale for children (WISC) (Wechsler *et al.*, 1992), which was designed as a measure of IQ for children between the ages of 6 and 16 years and is the most widely used cognitive test of its kind (Canivez and Watkins, 1998). The WISC has five verbal subtests and five performance subtests. The verbal subtests cover a pupil's knowledge, understanding of similarities, mental arithmetic, vocabulary and comprehension of situations. The performance subtests include spotting missing items in pictures, putting pictures in order, copying shapes, translating block designs into reality and completing other puzzles. The children were also given the forward and backward digit span task (which is a measure of short-term storage capacity), repeating lists of digits of differing lengths, firstly in the order in which they were presented and secondly in reverse (Northstone *et al.*, 2006). We create a total score from these WISC components, which we standardize on the basis of our common sample to have a mean of 0 and a standard deviation of 1.

As described above, our main identification strategy depends on a comparison of RDD estimates of the effect of being born just after rather than just before the academic year cut-off by using national achievement tests (sat on the same date) and those administered as part of the survey (sat at the same age). Importantly, for our purposes, the national achievement tests taken at age 7 years (which are also known as key stage (KS) 1) and certain questions used to create the WISC score are very similar indeed.

We report the correlation between scores for each component of KS 1 and IQ (WISC) measures of cognitive development in Table10, separately for children who were born in August (to the left of the academic year discontinuity in England) and September (to the right of the discontinuity). A high correlation indicates that both measures capture similar information about a child's development. The correlations between each KS 1 component and the WISC information (assessing knowledge, understanding similarities, mental arithmetic, vocabulary and digit span components) are high. Although other components (such as the comprehension score) are less highly correlated with the national achievement tests, this does not seem to affect the total score, which has the highest correlation of all. This suggests that the WISC and KS 1 scores contain similar information about a pupil's cognitive development, and therefore that our identification strategy is valid.

**Table 10:** Correlation between WISC (IQ), listening comprehension, oral expression and KS 1 scores†

*Score*	*KS 1:*	*KS 1:*	*KS 1:*	*KS 1:*
	*reading*	*writing*	*mathematics*	*points score*
*August-born children*				
WISC: information score	0.54	0.51	0.54	0.56
WISC: similarities score	0.41	0.37	0.41	0.42
WISC: arithmetic score	0.48	0.47	0.48	0.50
WISC: vocabulary score	0.40	0.41	0.40	0.42
WISC: comprehension score	0.18	0.17	0.18	0.19
WISC: digit span score	0.40	0.38	0.40	0.42
WISC: forward digit span score	0.32	0.30	0.32	0.33
WISC: backward digit span score	0.32	0.29	0.32	0.33
WISC: picture completion score	0.20	0.21	0.20	0.22
WISC: coding score	0.32	0.32	0.32	0.34
WISC: picture arrangement score	0.24	0.22	0.24	0.24
WISC: block design score	0.26	0.27	0.26	0.28
WISC: object assembly score	0.21	0.19	0.21	0.22
*WISC: total score*	0.57	0.55	0.57	0.59
WOLD: listening comprehension	0.32	0.29	0.32	0.33
WOLD: oral expression	0.46	0.39	0.46	0.46
*September-born children*				
WISC: information score	0.52	0.48	0.52	0.53
WISC: similarities score	0.34	0.33	0.34	0.36
WISC: arithmetic score	0.46	0.40	0.45	0.46
WISC: vocabulary score	0.36	0.36	0.36	0.38
WISC: comprehension score	0.21	0.20	0.21	0.21
WISC: digit span score	0.35	0.35	0.36	0.38
WISC: forward digit span score	0.27	0.27	0.27	0.29
WISC: backward digit span score	0.28	0.28	0.29	0.30
WISC: picture completion score	0.20	0.21	0.20	0.22
WISC: coding score	0.32	0.35	0.32	0.35
WISC: picture arrangement score	0.20	0.16	0.21	0.20
WISC: block design score	0.29	0.29	0.29	0.31
WISC: object assembly score	0.20	0.20	0.20	0.21
*WISC: total score*	0.56	0.54	0.56	0.59
WOLD: listening comprehension	0.25	0.22	0.25	0.25
WOLD: oral expression	0.44	0.44	0.43	0.46

† WOLD, Wechsler objective language dimensions.

For robustness we also present results where the dependent variable is constructed from the most highly correlated components of the WISC—namely those with a correlation above 0.3 in the whole sample (information, similarities, arithmetic, vocabulary, digit span, backward digit span and coding) and 0.4 in the whole sample (information, arithmetic and vocabulary). These results are presented alongside the WISC measure based on all components.

In addition, Table10 reports the correlation for alternative measures of cognitive ability, measured during the same assessment period as the WISC subtests: two subtests of the ‘Wechsler objective language dimensions’ (Rust, 1996), which have similarly high correlation with the KS 1 scores. In the listening comprehension subtest (which has a correlation of around 0.3 with KS 1 scores), the child listens to the tester read aloud a paragraph about a picture, which the child is shown. The child then answers questions on what they have heard. In the oral expression subtest (which has a correlation of around 0.4 with KS 1 scores), a series of pictures is shown to the child to assess their expressive vocabulary.

As we observe only measures of non-cognitive skills as part of the survey, we focus our attention here on estimating a combined age of starting school, length of schooling and relative age effect. We consider four different measures of non-cognitive skills or behaviours: scholastic competence, self-esteem, whether the child reports not liking school and locus of control.

Our scholastic competence and self-esteem measures are each created from six items of a shortened form of Harter's self-perception profile for children (Harter, 1985), which children were asked at age 8 years. They respond by ‘posting’ whether a series of statements (such as ‘Some children feel that they are very *good* at their school work’ and ‘Some children are *happy* with themselves as a person’) was true or not for them in a box. The score is standardized on the whole sample to have mean 0 and standard deviation 1.

Our indicator for a child not liking school is coded on the basis of a self-reported response of ‘not much’ or ‘no’ when asked whether they like school at age 8 years. Our locus-of-control measure is a total score from a shortened version of the Nowicki–Strickland internal–external scale (Nowicki and Strickland, 1973) which makes use of self-reported responses among preschool and primary age children, here administered at age 8 years. Locus of control captures the perception of the connection between one's actions and their consequences (Rotter, 1966), with a higher score indicating a more external locus of control (i.e. a lower belief that their own actions have consequences, and a stronger belief that fate or destiny is playing a role). The total score is standardized on our common sample to have mean 0 and standard deviation 1.

As described above, to improve the precision of our estimates, and to ensure that the individuals whom we are comparing are as similar as possible, we include all individual and family background characteristics that are listed in Table2 in our models (although their inclusion changes our results only marginally; see Tables5 and 6). See Crawford *et al*. (2011) for further information on how these variables were constructed.

### 3.2. Millennium Cohort Study

The MCS—which we use to carry out a robustness check on our main estimates—is a longitudinal study that has followed approximately 18500 children sampled from all live births in the UK between September 2000 and January 2002 (although we focus on the 5019 cohort members who were born in England between September 2000 and August 2001 and started school in an area in which the policy was for all children to start school in the September after they turn 4 years of age). The first MCS survey was conducted when the child was around 9 months old, with follow-ups to date at ages 3, 5 and 7 years (waves 1–4 respectively).

**Fig 2:**
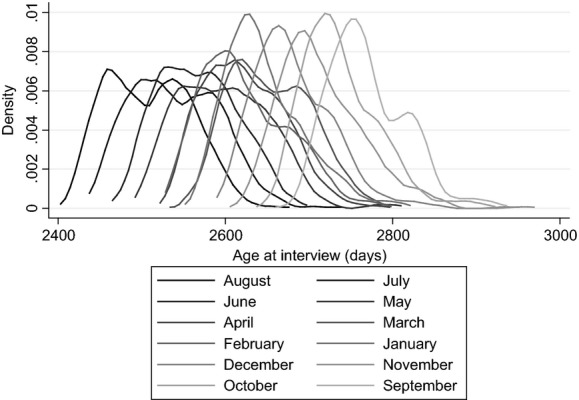
Overlap of age at MCS test: the sample includes only those living in local authorities that operated a single point of admission in the academic year in which the children started school; the key refers to the child's month of birth

MCS cohort members have been subjected to a number of tests of their cognitive skills, mostly using the British ability scales, which are a cognitive assessment battery that was designed for children aged between 3 and 17 years (Elliott, 1983). For comparability with the ALSPAC results we focus on the measure of cognitive ability at age 7 years, when cohort members were tested on word reading, pattern construction and mathematics. We sum the scores from all available tests and standardize these scores within cohort to have mean 0 and standard deviation 1. These tests were mostly taken within a window around the child's birthday, but there is some (random) variation: for example, Fig.2 shows the distribution of age at interview in wave 4 (around age 7 years) for MCS cohort members who were born in each month and provides evidence of a high, but not perfect, degree of overlap by month of birth.

**Fig 3:**
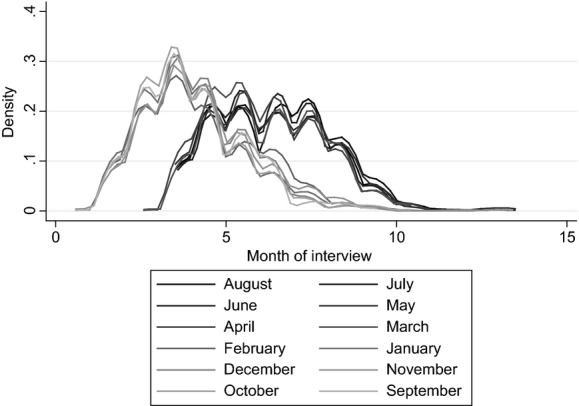
Overlap of month of MCS interview: the sample includes only those living in local authorities that operated a single point of admission in the academic year in which the children started school; the key refers to the child's month of birth

Similarly, Fig.3 shows the distribution of month of interview among children who were born in different months of the year for the same wave. Although, as is clear from Fig.3, the timing of the wave 4 survey was staggered so that those who were born between September 2000 and February 2001 would be interviewed earlier than those who were born between March and August 2001, the exact timing of this interview (and cognitive assessment) within these periods was effectively random. Moreover, some degree of common support in the month in which children were tested is evident for children who were born in all months of the year, even comparing across testing periods. This effectively allows us to use the same test to compare the differences with and without the age-at-test effect—the former including a control for the month in which children were interviewed, thus effectively mimicking the scenario in which all children were tested on the same date, and the latter including a control for the age at which children were interviewed, thus replicating the scenario in which all children were tested at the same age.

We use this approach as a robustness check on our main estimates, in which we must rely on the comparability of different tests taken at different times and in different scenarios to estimate an upper bound on the age-at-test effect. The assumptions underlying this secondary approach are different, but similarly stringent: the timing of the survey must be independent of underlying academic ability, conditional on pupils' month of birth and other observable characteristics, and we must assume that the effect of age and month of interview are linear to extrapolate across those who are born in different months of the year for which common support does not extend. The need to extrapolate outside the common support is fairly limited, however, particularly for month of interview (see Fig.3). Moreover, Crawford *et al*. (2013) also found similar results by using data from wave 3 (age 5 years), when children were surveyed much closer to their birthday, thus providing much greater overlap in age at interview (but less in month of interview).

## 4. Results

### 4.1. Cognitive skills

This section uses the regression discontinuity approach that was described above to document differences in various measures of cognitive skills among children who were born at the start and end of the academic year in England. In particular, it compares the differences in national achievement test scores (taken at a particular point in the academic year in which a child turns 7 years) with those (e.g. the WISC IQ score, and various measures of listening comprehension and oral expression) measured around the time of a child's eighth birthday as part of the ALSPAC survey to understand better the drivers of the differences that we observe. This analysis has important policy implications; is age normalizing test scores appropriate and sufficient to overcome observed differences in cognitive outcomes?

**Fig 4:**
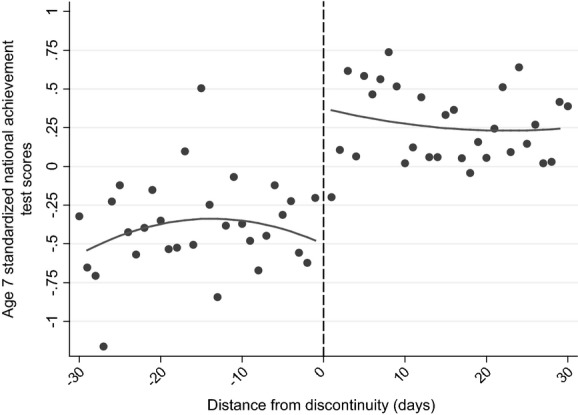
Discontinuity in KS 1 scores: a window of 30 days on either side of the discontinuity is applied: both discontinuities (across cohorts) have been pooled to increase sample sizes; the model is as specified in Section Methodology and identifying assumptions, omitting background characteristics; a common sample is imposed (●, average attainment for pupils born a certain distance from the discontinuity (in days))

Fig.4 shows the discontinuity in national test scores when September-born children are aged 7 years and 8 months and August-born children are aged 6 years and 9 months for those who were born up to 30 days on either side of the September 1st cut-off. It is clear that there is a large jump in test scores, with those marginally assigned to the treatment group (the oldest in the academic year) scoring, on average, over 0.8 standard deviations higher than the youngest in the academic year.

Columns (1) and (2) of Table11 present the corresponding RDD estimation results (without and with accounting for background characteristics). As described in Section Methodology and identifying assumptions, these models include a quadratic control for distance from the discontinuity (which is allowed to vary on either side of the discontinuity) and a range of individual and family background characteristics (where indicated). The estimated effect of being the oldest in the academic cohort (receiving the treatment) is around 0.8 standard deviations when pupils are around age 7 years, which confirms the graphical representation of the treatment effect around the discontinuity shown in Fig.4. The estimates are largely unchanged when background characteristics are included, which is consistent with our finding that there is no systematic relationship between distance from the discontinuity and parent or child characteristics in these data (presented in Tables4).

**Table 11:** RDD estimates: cognitive skills†

	*National achievement test scores, age 7 years*		*WISC, age 8 years*		*WISC: correlation above 0.3, age 8 years*		*WISC: correlation above 0.4, age 8 years*		*WOLD: comprehension, age 8 years*		*WOLD: expression, age 8 years*	
	*(1)*	*(2)*	*(3)*	*(4)*	*(5)*	*(6)*	*(7)*	*(8)*	*(9)*	*(10)*	*(11)*	*(12)*
Treatment effect	0.876‡	0.835‡	0.101	0.065	0.089	0.046	−0.011	−0.029	−0.170	−0.127	0.075	0.050
	(0.188)	(0.219)	(0.205)	(0.191)	(0.188)	(0.179)	(0.191)	(0.181)	(0.217)	(0.254)	(0.156)	(0.162)
Distance	Yes	Yes	Yes	Yes	Yes	Yes	Yes	Yes	Yes	Yes	Yes	Yes
Distance × treatment	Yes	Yes	Yes	Yes	Yes	Yes	Yes	Yes	Yes	Yes	Yes	Yes
Distance^2^	Yes	Yes	Yes	Yes	Yes	Yes	Yes	Yes	Yes	Yes	Yes	Yes
Distance^2^ × treatment	Yes	Yes	Yes	Yes	Yes	Yes	Yes	Yes	Yes	Yes	Yes	Yes
Background characteristics	No	Yes	No	Yes	No	Yes	No	Yes	No	Yes	No	Yes
*N*	982	982	982	982	982	982	982	982	982	982	982	982
*R*^2^	0.101	0.315	0.005	0.212	0.007	0.212	0.003	0.230	0.007	0.142	0.005	0.173

† Standard errors are robust and clustered by distance to the discontinuity. ‘Distance’ refers to the assignment variable: the distance from the discontinuity. The model is as specified above and includes a range of background characteristics. WISC components included where the correlation in the whole sample is above 0.3 are information, similarities, arithmetic, vocabulary, digit span, backward digit span and coding. WISC components included where the correlation in the whole sample is above 0.4 are information, arithmetic and vocabulary. WOLD, Wechsler objective language dimensions.

‡ *p* < 0.001.

**Fig 5:**
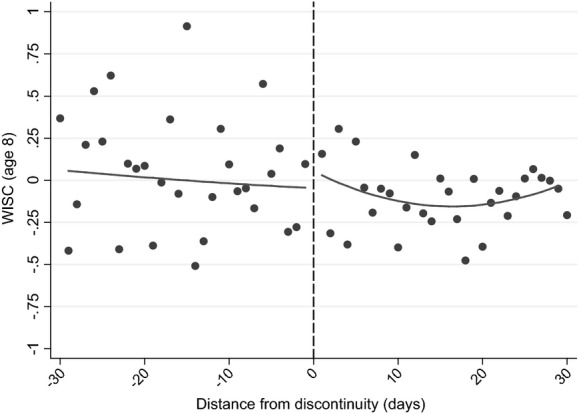
Discontinuity in WISC (IQ) standardized score (see the caption for Fig.4)

Fig.5 and columns (3) and (4) of Table11 move on to document differences in cognitive skills by using the WISC IQ score, measured around the time of the child's eighth birthday (Table11 showing the results without and with accounting for background characteristics). In comparison with tests that are nationally administered, where children are assessed on the same day rather than at the same age, these estimates are the result of differences in age of starting school, length of schooling and relative age, with the ‘age-at-test’ effect effectively eliminated.

Fig.5 shows that there is only a very small jump in test scores among children who were born just after the discontinuity, which columns (3) and (4) of Table11 suggest is not significantly different from 0. This is in marked contrast with the results from national achievement tests, which are also affected by the age at which a child sits the test. This suggests that either

(a) the age at which a child sits a test is the most important driver of the difference in outcomes for children who are the oldest and youngest in their cohort, or
(b) drivers of the differences in outcomes change significantly between age 7 and age 8 years or
(c) the WISC is not comparable with the KS tests.

Our judgement is that the first is the most likely explanation of our results. The second potential explanation seems highly unlikely, as Crawford *et al*. (2007, 2011) showed that there are significant differences between those who are born on either side of the discontinuity at older ages as well. The third potential explanation is feasible, as the WISC is designed to measure IQ rather than learned material, but it is unlikely that the differences in content are sufficient to remove the large significant difference that is observed at age 7 years. In particular, the evidence that was presented in Section Methodology and identifying assumptions suggests that these measures largely reflect similar measures of children's cognitive development. This conclusion is strengthened when we focus on differences in the components of the WISC that are most highly correlated with KS 1 scores: the results from these analyses (which are presented in columns (5)–(8) of Table11) suggest that there are no significant treatment effects, as for our main results. In addition, using alternative measures of cognitive development that were assessed on the same day as the WISC components (and have a similarly high correlation with KS 1 scores) show no significant treatment effects either (presented in columns (9)–(12) of Table11).

These results are also robust to variation in the size of window around the discontinuity and in the specification of the polynomials in distance from the discontinuity (both presented in Table5). For example, the estimate of the treatment effect for KS 1 scores varies from 0.83 standard deviations in the main specification to 0.70 standard deviations when a window of 20 days is imposed, 0.77 when a window of 40 days is imposed, 0.77 when distance is entered linearly and 0.59 when distance is cubed. These estimates are not significantly different from one another. Table5 also shows that results are not significantly different from one another when we impose different sample restrictions (either imposing no common sample or a sample that has at least one—but not necessarily all—outcomes observed in the ALSPAC), when we impose a common trend across the discontinuity, or when distance from the discontinuity is grouped into 2-day bins. Excluding those who are born very close to the discontinuity—within 5 days either side—increases the size of the estimate at KS 1, suggesting that there may be some measurement error in the assignment variable, although the standard errors also increase, meaning that this estimate is still not significantly different from our main treatment effect. Table5 also shows that the standard errors are broadly similar if we cluster at the school level rather than by distance to the discontinuity.

We also ran a series of placebo tests (which are presented in Tables9) in which we estimate the effect of being born just after rather than just before a series of alternative discontinuities which should in principle have no bearing on the age at which children start school or sit tests, or in how old they are relative to their classroom peers. These results show that the only significant difference in terms of national achievement test scores occurs at the September 1st discontinuity.

In contrast with assessments that are taken on the same day, the estimates for assessments taken at around the same age are consistently small and not significantly different from 0, in all robustness checks undertaken.

These findings show a stark and robust difference between the RDD treatment effects that are estimated by using assessments taken at the same age and on the same date. Although a direct comparison between the two estimates enables us to identify only an upper bound on the age-at-test effect (as length of schooling will also differ between the two), it seems unlikely that the close-to-zero treatment effect that is estimated by using tests taken at around the same age is hiding a large and significant positive length of schooling effect. This suggests that it is the age-at-test effect that primarily drives the differences in national achievement test scores that were found between children who were born at the start and end of the academic year.

This corroborates evidence that has been found elsewhere (e.g. Crawford *et al*. (2007), Smith (2010) and Black *et al*. (2011)) and suggests that one simple solution to the penalty that is faced by those born at the end of the academic year would be to normalize national achievement test scores by age: this would mean being judged relative to others their own age, rather than others of potentially quite different ages within their class or school year group, thus providing a fairer comparison between the abilities and potential of summer-born children and their peers.

These results may be subject to criticism that tests that are taken outside school are different from tests that are taken in school, however. For example, a child who is assessed outside school may be subject to less stereotype threat, in which performance can be negatively affected by the experience of anxiety or concern where a person has the potential to confirm a negative stereotype about their social group. Stereotype threat may therefore be heightened in classroom situations, which would negatively affect the performance of the youngest in the year. This may mean that the relative age effect, for example, is not constant across the two assessments.

To address this criticism we additionally present analysis that compares the performance of those who were born at the start and end of the academic year when we replicate the situation in which tests are taken at the same point in time (where the age-at-test effect is present) and at the same age (thus eliminating the age-at-test effect). As discussed above, this is possible through exogenous variation in the timing of assessments of children participating in the MCS. Unfortunately an RDD design is not possible here, as the MCS sample in England were born within a single academic cohort; the treatment effect described below is therefore interpreted as the within-cohort difference, on average, between those who are the oldest (born in September) and youngest (born in August) in their academic year.

**Fig 6:**
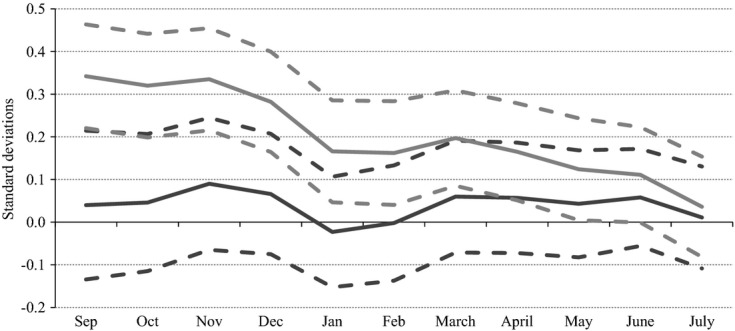
Difference in British ability scores at age 7 years, when measured at the same age or same point in time (the sample includes only those living in local authorities that operated a single point of admission in the academic year that the children started school; both sets of estimates account for a variety of individual and family background characteristics): 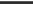, differences, on average, between those born in August and those born in other months of the year, accounting for age at test to mimic the case when children are tested at around the same age; 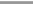, differences controlling for date of interview, thus mimicking the results of a test taken at the same point in time; 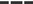, 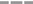, 95% confidence interval around the estimates

Table12 presents the results from this final robustness check (which are also presented graphically in Fig.6). It is clear that, when we replicate the situation in which children who are born in different months are assessed at the same age, the difference in performance between those who are the oldest and youngest in the academic year is close to (and not significantly different from) 0. When we replicate the situation in which children are assessed at the same point in time, however, the difference becomes larger and is significantly different from 0, at around 0.34 standard deviations.

**Table 12:** Results from alternative methodology†

	*Results for replicate*		*Results for replicate*	
	*test on birthday*		*test on same day*	
September	0.040	0.089	0.342‡	0.062
October	0.046	0.082	0.320‡	0.062
November	0.090	0.079	0.335‡	0.061
December	0.066	0.072	0.282‡	0.060
January	−0.023	0.066	0.166§	0.061
February	−0.002	0.069	0.162§	0.062
March	0.060	0.067	0.197‡	0.057
April	0.057	0.066	0.165§	0.058
May	0.043	0.064	0.124§§	0.061
June	0.058	0.058	0.111	0.057
July	0.011	0.061	0.036	0.060
Parents' characteristics	Yes		Yes	
Age at interview	Yes		No	
Month of interview	No		Yes	
*N*	4220		4220	

† The sample includes only those living in local authorities that operated a single point of admission in the academic year that the children started school.

‡ *p*< 0.001.

§ *p*< 0.01.

§§ *p*< 0.05.

This difference is smaller than the differences in KS 1 scores observed at the same age and shown in Table11; this may be a result of the different methodology employed, the different sample of pupils used or the different tests taken. Nonetheless, these estimates strengthen our previous conclusion: differences in cognitive assessments between those who are born at the start and end of the academic year seem to be largely driven by differences in the age at which children sit the test, although again we cannot rule out that length of schooling may be playing a small role as well.

### 4.2. Non-cognitive skills

**Fig 7:**
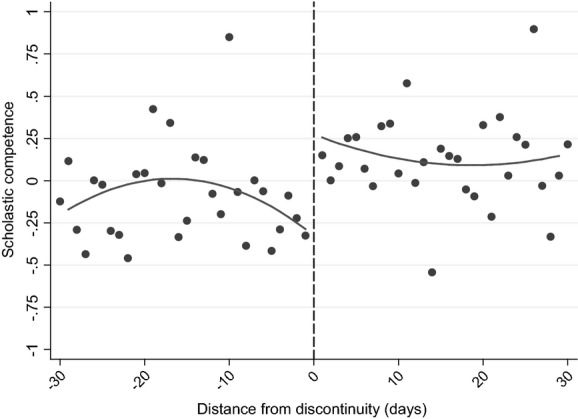
Discontinuity in scholastic competence (reported by ALSPAC member) (see the caption for Fig.4)

**Fig 8:**
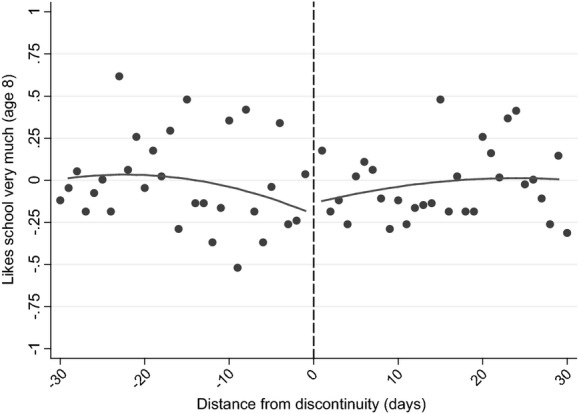
Discontinuity in whether the child likes school very much (see the caption for Fig.4)

**Fig 9:**
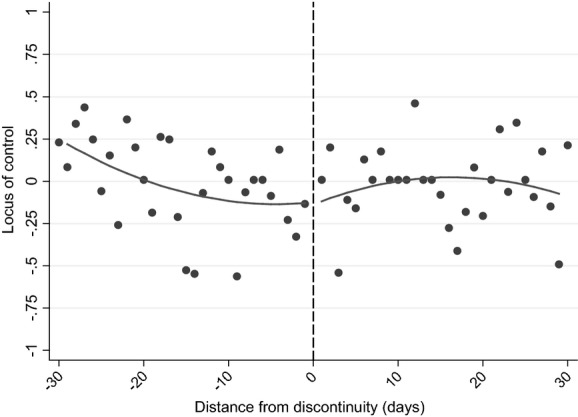
Discontinuity in locus of control (reported by ALSPAC cohort) (see the caption for Fig.4): the scale of locus of control moves from more internal to more external (less belief that they can control the events that affect them)

**Fig 10:**
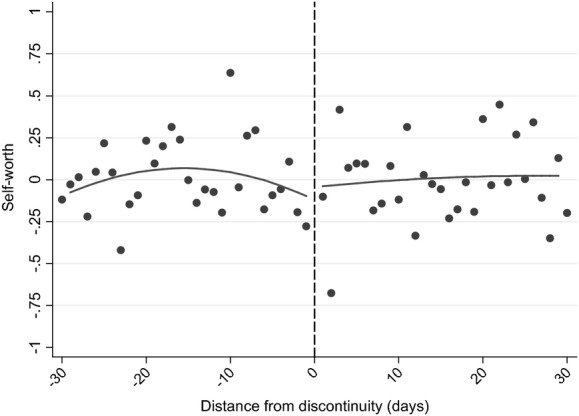
Discontinuity in global self-worth (reported by ALSPAC cohort member)

The results that were presented above have shown that there is little difference in cognitive test scores between children who are born at the start and end of the academic year when they are assessed at the same age, suggesting that a policy of age normalizing test scores might be sufficient to overcome the disadvantages that summer-born children in England face. This section considers whether the same is also true for some wider measures of skills, including the child's own assessment of their scholastic competence (Fig.7) and whether they like school (Fig.8), their locus of control (i.e. how in control they feel of their own destiny) (Fig.9) and global self-worth (Fig.10). The RDD estimates and their significance are shown in Table13.

**Table 13:** RDD estimates: non-cognitive skills†

	*Results for scholastic competence, age 8 years*		*Results for likes school very much, age 8 years*		*Results for locus of of control, age 8 years*		*Results for self-esteem, age 8 years*	
	*(1)*	*(2)*	*(3)*	*(4)*	*(5)*	*(6)*	*(7)*	*(8)*
Treatment effect	0.557‡	0.382§	0.051	−0.006	−0.028	−0.149	0.042	−0.032
	(0.180)	(0.179)	(0.181)	(0.184)	(0.192)	(0.197)	(0.222)	(0.224)
Distance	Yes	Yes	Yes	Yes	Yes	Yes	Yes	Yes
Distance × treatment	Yes	Yes	Yes	Yes	Yes	Yes	Yes	Yes
Distance^2^	Yes	Yes	Yes	Yes	Yes	Yes	Yes	Yes
Distance^2^ × treatment	Yes	Yes	Yes	Yes	Yes	Yes	Yes	Yes
Background characteristics	Yes	Yes	Yes	Yes	Yes	Yes	Yes	Yes
Academic attainment	No	Yes	No	Yes	No	Yes	No	Yes
*N*	982	982	982	982	982	982	982	982
*R*^2^	0.098	0.132	0.078	0.081	0.147	0.163	0.072	0.078

† See the notes to Table11. Academic attainment refers to KS 1 national assessment scores, taken at the end of the previous academic year. The scale of locus of control moves from more internal to more external (less belief that they can control the events that affect them).

‡ *p*< 0.01

§ *p*< 0.05.

Fig.7 and column (1) of Table (13) show that there is a sizable and significant difference between children who are born at the start and end of the academic year in terms of their view of their own scholastic competence at age 8 years, with those who are born just after the discontinuity scoring around 0.6 standard deviations higher than those who are born just before the discontinuity. This holds regardless of whether background characteristics are accounted for. This difference is large: around 70% of the effect size that was found for national achievement test scores at age 7 years and three times as large as the difference in scholastic competence that was observed between children who were born to households with the highest and lowest levels of income (in the same sample of children) (although the robustness checks that are presented in Table6 suggest some variation: between around 0.3 and 0.7 standard deviations).

Column (2) of Table13 suggests that children who are born at the start of the academic year continue to have a more positive view of their own scholastic competence than children who are born at the end of the academic year, even after conditioning on their attainment in national KS 1 assessments. The coefficient is reduced slightly but is not significantly different from the coefficient from the specification without accounting for academic attainment. This suggests that the differences in perceived scholastic competence between those who are born at the start and end of the academic year may be due in part to the information that children receive from national assessments, but they are also likely to be influenced by classroom interactions and other assessments.

By contrast, Figs 8–10 and columns (3), (5) and (7) of Table13 illustrate that there are no significant differences between children who are born on either side of the discontinuity in terms of their self-esteem, their locus of control or whether they like school, although the treatment effects for global self-worth and liking school are positive. Again, this holds regardless of whether background characteristics or prior attainment at KS 1 are accounted for, although each estimated treatment effect is slightly lower when accounting for prior attainment. This suggests that children's enjoyment of school and wider feelings of self-worth are not being significantly adversely affected by their academic performance, although higher academic attainment in school does seem to translate into higher scholastic competence. This is consistent with wider literature that self-esteem is influenced by several factors, including, but limited to, academic ability (Harter, 1993) and in adolescents seems to be largely determined by background characteristics (Bachman *et al.*, 1977; Maruyama *et al.*, 1981).

These results suggest that some combination of relative age, length of schooling and/or the age at which children start school have a significant negative effect on those who are born younger in the academic year in terms of their perceptions of their own scholastic self-competence, which is not driven by the age at which they are asked the questions. In contrast with the results for cognitive skills, therefore, these results suggest that a policy response of appropriately age adjusting tests may not completely resolve the issues that are associated with being born later in the academic year in terms of these wider measures of skills: although it is plausible that the scholastic confidence of younger children may respond positively to feedback being provided by using appropriately age-adjusted test scores, the fact that there remains a significant difference in this outcome between children who are born at different times of the year even when it is asked at the same age suggests that other factors may also be playing a role here.

## 5. Conclusions

This paper has built on the existing literature in England by using a more robust RDD to confirm that there are large and significant differences between children who are born at the start and end of the academic year (September and August respectively in England) in terms of their cognitive skills when measured by using national achievement tests. More importantly, it has built on the existing international literature by using two complementary identification strategies to investigate what drives these differences in cognitive outcomes and has also provided the first evidence on whether the same mechanisms drive the differences in non-cognitive skills that are observed between children who are born at the start and end of the academic year.

To do so, we compare differences in nationally set and administered achievement tests (which are completed on the same day) with differences in other, similar, cognitive outcomes which are assessed as part of a survey at around the same age, thus effectively eliminating the difference in age at test between children who are born just before and just after the academic year cut-off. This allows us to understand to what extent the combined effects of relative age, length of schooling and age of starting school are responsible for the differences in cognitive and non-cognitive skills that we observe between children who are born at the start and end of the academic year.

In contrast with the results by using tests measured on the same date, we find no differences in cognitive development between children who are born just before and just after the academic year cut-off when tests are taken at around the same age. This leads us to conclude that the combination of the age of starting school, length of schooling and relative age effects does not have a significant effect on cognitive development, and thus that age at test is the most important factor driving the difference between the oldest and youngest children in an academic cohort. This finding confirms earlier work using a parametric approach (Crawford *et al.*, 2007) and is replicated in our supplementary results by using a different identification strategy in a different source of data in which some children who are born in the same month are tested (by using the same test) at different points in time.

An appropriate policy response to the difference in assessments taken in schools would therefore be to adjust nationally set and administered tests appropriately by age and to provide feedback on the basis of these adjusted scores. But would this solution address potential differences in children's wider development as well?

We find no evidence that children's locus of control, self-assessed global self-worth or enjoyment of school are significantly different when these skills are measured around the child's eighth birthday. However, we find strong evidence that a child's view of their own scholastic competence is significantly different between children who are born on either side of the discontinuity, even when these questions are asked at around the same age, thus eliminating the age-at-test effect. This means that other policy responses may be required to overcome the differences in perceived scholastic confidence among children who are born in different months of the year—but not necessarily so: it may be that children's views of their own scholastic competence would change in response to the introduction of appropriately age-adjusted tests, with feedback provided relative to others their own age rather than those in their class or school year group.
